# Lower Income Levels in Australia Are Strongly Associated With Elevated Psychological Distress: Implications for Healthcare and Other Policy Areas

**DOI:** 10.3389/fpsyt.2018.00536

**Published:** 2018-10-26

**Authors:** Anton N. Isaacs, Joanne Enticott, Graham Meadows, Brett Inder

**Affiliations:** ^1^School of Rural Health, Latrobe Valley and West Gippsland, Monash University, Traralgon, VIC, Australia; ^2^Department of General Practice, School of Primary and Allied Health Care, Monash University, Notting Hill, VIC, Australia; ^3^Southern Synergy, Department of Psychiatry, School of Clinical Sciences, Monash Health, Monash University, Dandenong, VIC, Australia; ^4^Australian NHMRC Centre of Research Excellence in Suicide Prevention, Brain and Mind Centre, University of Sydney, Camperdown, NSW, Australia; ^5^Department of Econometrics & Business Statistics, Monash Business School, Monash University, Caulfield, VIC, Australia

**Keywords:** income inequality, psychological distress, mental disorders, health status indicators, socioeconomic factors, poverty, health surveys, health care evaluation mechanisms

## Abstract

**Background:** Australia is a high-income country with increasing income inequality. It is unclear whether Australia's well-developed mental healthcare system is making a difference to population mental health and the Federal Government has targeted outcomes accountability in service funding strategies. In high-income countries, evidence generally suggests that income inequalities increase mental disorders among the poor. This study examined psychological-distress rates—a marker of mental ill- health—as varying by income among Australians living within and outside of capital cities.

**Methods:** Secondary data analysis was undertaken using the population-level mental health indicator of the Kessler Psychological Distress Scale (K10) as reported for 12,332 adults in the 2011/2012 National Health Survey (NHS) of Australia. K10 scores of 22 and higher indicated high/very-high distress, and 30 and higher denoted very-high distress. Very-high distress levels are strongly predictive of serious mental illness.

**Results:** Among the poorest one-fifth of Australians, 1 in 4 people have psychological distress at a high/very-high level; this compares to about 1 in 20 people in the richest one-fifth of Australians. About 1-in-10 people making up the poorest one-fifth of Australians have current very-high distress, and this reduces to <1-in-50 people in the richest one-fifth. These disparities are consistent both within and outside of capital cities. The national prevalence of elevated distress within income quintiles varies greatly, with Poor/Rich Quintile Ratios of typically 4–5 for high/very-high levels and 7–8 for very-high levels. These effects operate more powerfully in areas marked by higher scores on the Index of Relative Socioeconomic Disadvantage.

**Conclusions:** Altering the strong association of lower income levels in Australia with elevated psychological distress would require a multi-dimensional social policy and healthcare approach. To assess the effectiveness of adopted strategies, population level indicators need to be developed with regular data-collection. The Poor/Rich quintile ratio (P/R QR) for high/very high K10 scores is a potential candidate for a mental health inequality outcome indicator since it is easily calculated from data obtained from a regularly conducted national survey, is easily understood and resonates with a wider audience. Further research on the development of such indicators is also needed.

## Introduction

It is well known that social and economic disadvantage increases the risk of mental disorders and its adverse consequences. Hence, the WHO Mental Health Action Plan 2013–2020 calls upon states to focus more on disadvantaged groups (1). Poverty or income disadvantage has been shown to be directly related to psychological distress and mental health (2). Poorer communities are more frequently exposed to risk factors for psychological distress and mental disorders such as violence, crime, social conflict, civil unrest, homelessness and unemployment. Poor communities also tend to have far worse consequences of mental disorders than more well-to-do communities (3).

Financial hardship, sometimes referred to as “financial stress” or “financial distress” refers to the reaction to one's poor financial well-being which includes one's inability to pay bills and repay debts (4). Financial hardship and unsecured debt have been shown to be strongly associated with depression (5, 6) suicide, drug dependence, and psychotic disorders (7) and the likelihood of developing a mental disorder appears to increase with the amount of unpaid debt (8). On the other hand, when financial problems are addressed, symptoms of psychological distress tend to decrease (9, 10) and those with severe mental disorders and a better financial status tend to have fewer psychiatric symptoms and quality of life (11, 12).

Income inequality refers to the degree of uneven distribution of income within a population. When income inequality increases, distress levels among those at the lower end of the economic ladder tend to increase. Population based studies from Canada (13), United Kingdom (14), and the United States (15) indicate that income inequality increases the risk of mental disorders among the poor in high-income countries. Furthermore, a 9-year longitudinal study of 30,000 UK parents (14) showed that rates of psychological distress were significantly higher for the lowest levels of absolute income. Not only are the lowest income earners affected in unequal societies but also the entire population. Pickett and Wilkinson (16) show that the more inequality present in a country, the higher the rates of mental illness in the population and vice versa. Furthermore, Cummins argues that the relationship between income and wellbeing is not linear (17). If people are poor, then a better income could relieve their distress. However, over a threshold level of income that takes care of their day to day needs, an increase in income does not increase their subjective wellbeing (17). Once income levels drop to below this threshold, there appears to be a linear association between income and subjective wellbeing.

Australia is a high-income country with a well-developed mental healthcare system that in policy terms is described as providing coverage “for all Australians” (18). Population studies of mental healthcare delivery have however identified substantial and unevenly distributed treatment gaps. The 2007 National Survey of Mental Health and Wellbeing (NSMHW) in Australia showed that 1-in-5 persons had a mental disorder in the previous year (19) and between 35 and 50% of people affected by mental illness received no treatment for their disorder (19). The “Universal” coverage of the National Health Insurance Scheme, “Medicare” whereby professional services can be supported by patient-assigned rebates (20) does not translate into equity of access to services funded through this route. For example, increasing remoteness is associated with lower service activity (21): the annual rate (per 1,000 population) for Medicare-subsidized mental health-specific services delivered by GPs is 76–79 in major cities and inner regional areas, reducing to 50 and 25 in outer regional and remote areas, respectively. For psychiatrists the rate is 92 in major cities reducing to 37 in inner regional areas, and then 13 and 4 in outer regional and remote areas, respectively (21). Furthermore, much of the treatment delivered for mental health problems does not follow the standards for clinical practice guidelines and is not targeted to those who are in most need (22). As a result, despite an increased use of antidepressants, an increased workforce as well as psychological therapies, the prevalence of mental disorders appears to be on the rise (22).

Rurality does not appear to significantly influence the mental health status of people in Australia (23–25). Research from the Australian Rural Mental Health Study also showed little or no difference in levels of psychological distress between those living in rural and those in remote areas (26, 27).

The need to establish genuine accountability for mental health in Australia was recognized as early as 1992 with Australia's first National Mental Health Policy (28). Australia also implemented national and state/territory strategies that aim to improve the delivery and funding of mental health-related services (29). In order to provide a measure of performance and progress in the delivery of services, the Australian Government reports on two sets of indicators (30). However, these indicators do not allow one to assess whether the overall mental health of the population is improving or whether there are sections of the population that are experiencing an unmet need.

Indicators such as “Health service access,” “Health worker density and distribution,” and “Coverage of services for severe mental health disorders” are already included in the Global Reference List of 100 Core Health Indicators, 2015 of the World Health Organization (31). The need to develop and use indicators of mental health is now even more urgent given the Federal government's move to focus more on improving outcomes rather than increasing financial input into a system that may be dysfunctional (32). Indicators developed from population-level data assists policy-makers to design public health innovations and targeted interventions, (25) the importance of which has also been highlighted in the World Health Organization's Global Mental Health Action Plan 2013–2020 (1).

The Kessler Psychological Distress Scale (K10) was originally designed to measure psychological distress in populations (33, 34) but was also found to have better overall discriminatory power than the GHQ-12 in detecting DSM-IV depressive and anxiety disorders (35). In Australia, it has established associations of increasing score levels with increased prevalence of mental disorders (36). In the 2007 National Survey of Mental Health and Wellbeing across Australia, 79.6% of people with a K10 score in the very-high distress range (scores of 30 and above) had a 12-month CIDI (Composite International Diagnostic Interview) interview assessed mental disorder. Only 10.9% of those in the low category (scores of 15 or less) had such a disorder (36). The K10 instrument is therefore considered internationally to be appropriate to estimate the needs of the population for community mental health services, as anxiety and depression are the high prevalence conditions.

Many general practitioners (GPs) across Australia are already familiar with this scale (34). As part of a Medicare subsidized mental health care plan or review, a GP may ask a patient to complete the K10 as it measures previous 30-day distress, particularly symptoms of anxiety and depression (36). An individual's K10 score can assist the GP with the assessment of a mental disorder since higher scores are associated with increasing prevalence of mental disorders (34, 36). In addition, samples of nationally representative K10 scores also assist policy-makers with determining the population mental health status (19, 36, 37).

We aimed to explore how rates of psychological distress as measured by the K10 varied by income and geographic location (within and outside capital cities). We propose that comparing K10 scores across different household income groups might lead to meaningful insights regarding optimal mental healthcare delivery, with implications for state/territory and national health policy. We examine three hypotheses: First, that substantially higher rates of elevated distress are associated with lower household income in an Australian context. Second, that rates of elevated distress are lower among those living in a capital city compared to those residing outside of capital cities. Third, in line with previous research suggesting that the relationship between income and wellbeing is not linear, the principle of diminishing marginal returns suggests that there is a smaller difference in rates of elevated distress when comparing households in the richest Australian quintile and the second richest quintile, relative to the difference in rates between the second poorest quintile and the poorest quintile.

## Materials and methods

### Design

Secondary data analysis using the 2011/2012 National Health Survey (NHS), extend a previous population-level study (25) which compares rates of distress in populations living in capital cities to those outside capital cities. Data were collected for each survey by trained interviewers from the Australian Bureau of Statistics (ABS) (37). The 2011/2012 NHS involved a sample of 20,426 Australians; the K10 was applied with *n* = 15,381 of whom *n* = 12,332 were aged 18–64 years. The response rate was 84.8%. As comprehensively described elsewhere, sampling strategies were designed to provide representative estimates for Australia and some sub-populations including State/Territory, Capital Cities/other (37). Derived estimates from the survey are not applicable for those living in very remote areas of Australia or living in non-private dwellings because both of these factors were out of scope of the sample design.

### Measure: K10

The K10, a self-administered 10-item Likert format scale, measures psychological distress experienced in the past 30 days (34, 36). K10 scores ranged between 10 and 50, and we used score bands of low (10–15), moderate (16–21), high (22–29), and very-high (30–50) (34, 36). We created a combined high/very-high category with scores 22 and higher (25).

### Geographical, household income, and socioeconomic index of areas variables

We examined Greater Capital City Statistical Areas (GCCSA) with categories of Capital Cities and Balance of State (38). We also examined the 2011 Index of Relative Socioeconomic Disadvantage (IRSD) for areas, which is a composite index based on census information regarding socioeconomic factors and resources within an area (39). We examined household income using the average equalized gross weekly income and reported deciles (39). As with the IRSD, we aggregated this information into quintiles for use in the analyses. The rationale for doing this was similar to that for using IRSD quintiles; cell counts within deciles were sometimes small (e.g., count in the very-high K10 band in the top income decile was only 14).

### Statistical analyses

We calculated national representative estimates using data from adults aged 18–64 years in the NHS survey. All regressions were adjusted for sex and age group (years) using age categories: 18–24, 25–34, 35–44, 45–54, and 55–64 years. Survey weights provided by the ABS data custodians, which were calculated to account for survey design features, were used in all analyses to provide accurate estimation of statistics for the Australian population. Replicate weights, also provided by the ABS, were used for the calculation of design error. Information available from the ABS on the survey includes sampling strategy and sample description (40). Access to the survey Confidentialised unit record files and replicate weights was done by using the ABS Remote Access Data Laboratory (RADL) platform (37). Analyses were done by using this RADL platform using Stata version 11.0 (StataCorp LP, College Station, TX).

Level of distress was determined by K10 band scores. We reported percentages of people with very-high and high/very-high distress, subsequently stratified by sex, age group, Capital Cities/Other, IRSD quintile, and household income quintile. We used logistic regressions to adjust for age group and sex to determine odds ratios (ORs) for high/very-high distress according to IRSD quintile and household income quintile. This was done separately for samples defined by place of residence as Capital Cities or not.

A robustness check of the findings was conducted by repeating the analyses with the above mentioned variables plus interaction terms for two variables: IRSD and household income. Multiple interaction terms were investigated in the model because the IRSD and income variables were categorical data (quintiles 1, 2, 3, 4, and 5), and since quintile data has an ordinal structure then a total of 14 interaction terms were investigated. This was done to investigate if these two socioeconomic variables differentially interacted with each other.

Equity analyses consisted of calculating poor to rich quintile ratios and concentration indices. Poor/Rich Quintile Ratios were calculated as the ratio of elevated distress prevalence of the poorest (or most disadvantaged) quintile to the richest (or least disadvantaged) quintile. To measure inequity, we determined concentration indices (21, 25, 41) which lie between −1 and +1. We inspected each concentration curve and noted if the curve crossed the 45 degree equity line as this can lead to misleading concentration index values (21, 25). Negative indices represented greater prevalence in lower income households. We followed a convention of using an index threshold of ±0.2 as indicating a high level of inequality. An index of −0.2 would result from the poorest half of the population having 50% higher prevalence of distress than the richest half.

### Ethics approval

This study was exempted from ethics review by Monash University Human Research Ethics Committee because the non-identifiable data satisfied the National Statement on Ethical Conduct in Human Research.

## Results

There were *n* = 6,904 adults residing in capital cities, and *n* = 5,428 outside capital cities. Percentages of very-high distress and combined high/very-high distress appeared similar in both regions –high/very-high distress prevalence was 11.0% (95% CI = [10.2, 11.8]) in capital cities and 11.4% (95% CI = [10.2, 12.5]) outside capital cities, see Table 1.

**Table 1:** Prevalence of psychological distress across Australia, separated by Capital Cities and Balance of State.

**Number of 18–64 year olds**		**Greater capital city statistical areas ASGS 2011**							
		**K10 high/very-high distress**				**K10 very-high distress**			
		**Capital cities**		**Balance of state**		**Capital cities**		**Balance of state**	
		***n*** = **6,904**		***n*** = **5,428**		***n*** = **6,904**		***n*** = **5,428**	
		**% (95% CI)**		**% (95% CI)**		**% (95% CI)**		**% (95% CI)**	
Australian population		11.0	(10.2, 11.8)	11.4	(10.2, 12.5)	3.4	(2.9, 3.9)	4.0	(3.3, 4.6)
Gender	Male	8.8	(7.6, 9.9)	9.9	(8.3, 11.5)	2.5	(1.9, 3.2)	3.8	(2.6, 4.9)
	Female	13.3	(12.0, 14.6)	12.8	(11.1, 14.6)	4.3	(3.4, 5.1)	4.1	(3.2, 5.1)
Age group (years)	18–24	12.2	(9.9, 14.6)	11.4	(7.7, 15.1)	2.5	(1.1, 3.9)	4.1	(1.8, 6.3)
	25–34	11.4	(9.6, 13.2)	9.9	(7.5, 12.2)	3.2	(2.2, 4.2)	2.0	(0.8, 3.2)
	35–44	10.3	(8.7, 11.9)	12.3	(9.9, 14.7)	2.8	(2.0, 3.6)	4.1	(2.3, 5.9)
	45–54	11.3	(9.6, 13.0)	11.8	(9.3, 14.2)	5.1	(3.9, 6.3)	5.4	(3.6, 7.1)
	55–64	9.9	(8.0, 11.7)	11.4	(9.1, 13.8)	3.4	(2.5, 4.3)	4.0	(3.6, 5.5)
Income quintiles	(rich) 1	5.2	(4.0, 6.5)	6.5	(4.3, 8.7)	1.3	(0.7, 2.0)	1.6	(0.5, 2.8)
	2	8.4	(6.7, 10.1) ^d^	4.7	(3.1, 6.3)	2.2	(1.0, 3.3)	0.8	(0.3, 1.4)
	3	10.8	(8.7, 12.8) ^d^	8.8	(6.5, 11.0)	2.6	(1.5, 3.7)	3.9	(2.2, 5.5)
	4	17.4	(14.5, 20.4) ^d^	17.8	(14.7, 21.0) ^d^	6.7	(5.0, 8.5) ^d^	6.9	(4.7, 9.0) ^d^
	(poor) 5	24.9	(20.9, 28.8) ^d^	28.4	(22.9, 33.9) ^d^	9.7	(7.5, 12.0) ^d^	12.8	(9.0, 16.6) ^d^
	P for trend	<0.0001		0.04		<0.0001		0.04	
Poor/Rich quintile ratio		4.8	(4.0, 5.5)	4.4	(3.5, 5.2)	7.2	(5.6, 8.9)	7.8	(5.5, 10.1)
Concentration index		−0.29	(−0.21, −0.37)*	−0.34	(−0.17, −0.52)*	−0.38	(−0.23, −0.53)*	−0.44	(−0.24, −0.64)*
IRSD quintiles	(rich) 1	7.0	(5.4, 8.5)	6.5	(3.9, 9.2)	1.6	(0.9, 2.2)	2.5	(0, 4.9)
	2	8.9	(7.3, 10.5)	7.0	(4.4, 9.6)	3.1	(2.2, 4.0)	2.9	(0.9, 5.0)
	3	12.1	(10.1, 14.2) ^d^	11.7	(9.2, 14.3)	3.9	(2.4, 5.3) ^d^	3.5	(2.0, 4.9)
	4	14.3	(11.9, 16.7) ^d^	12.0	(9.9, 14.1) ^d^	4.5	(3.0, 6.0) ^d^	3.6	(2.4, 4.7)
	(poor) 5	16.9	(14.1, 19.7) ^d^	15.2	(12.0, 18.4) ^d^	5.4	(3.5, 7.4) ^d^	6.1	(4.6, 7.7)
	P for trend	<0.0001		<0.0001		<0.0001		<0.0001	
Poor/Rich quintile ratio		2.4	(2.0, 2.8)	2.3	(1.8, 2.8)		3.4 (2.2, 4.6)	2.5	(1.9, 3.1)
Concentration index		−0.17 (−0.16, −0.18)*		−0.12 (−0.43, 0.20)		−0.19 (−0.16, −0.23)*		−0.17 (−0.07, −0.27)*	

IRSD, Index of Relative Socioeconomic Disadvantage; CI, confidence interval; ABS, Australian Bureau of Statistics; K10, Kessler 10. The K10 very-high distress category represents a score of 30 or higher. Combined high/very-high distress category represents a score of 22 or higher. CI, Confidence Intervals are based on replication-based standard error estimation. ABS weighting was used to produce population estimates. Equity analyses are the Concentration indices and the Poor/Rich Quintile Ratios. *Significantly different from equity line.

d *K10 percentages had significant differences between quintile 1 and the quintile indicated (p<0.05), indicating less distress in quintile 1 (richest areas of Australia)*.

In households having the highest household incomes (income quintile 1), the prevalence of high level or greater distress and very high distress were significantly lower than in poorer households (income quintiles 2, 3, 4, or 5; see Table 1). Similarly, in advantaged regions with one-fifth of the population with the least disadvantage (IRSD quintile 1), the prevalence of distress were significantly lower than more disadvantaged areas (IRSD quintiles 3, 4 or 5; see Table 1). Regardless of residential status being inside capital cities or outside, the pattern of distress was similar by area disadvantage (IRSD quintile) and household income (income quintile), see Table 1.

The inclusion into the multivariate model of interaction terms for the two socioeconomic variables (IRSD quintile and household income quintile) produced non-significant interactions and the magnitude of the Odds Ratio for the other variables differed only minimally compared to the model without the interactions. This showed that IRSD and income factors are both associated with distress. The magnitudes of the Odds Ratios describe the poorest results (i.e., the greatest likelihood of elevated distress) for people living in the most disadvantaged IRSD quintile who have the lowest household incomes. The inequity across the socioeconomic gradient was much steeper by household income than IRSD disadvantage of areas, see Table 2. For example, compared to the most affluent quintile in capital cities, the most disadvantaged (IRSD) areas had a relative odds for high/very-high distress at 2.7 (95% CI = [1.9, 3.9]), whereas the poorest household income relative odds was much greater at 6.1 (95% CI = [4.4, 8.6]).

**Table 2:** Odds ratios (OR) for high/very high distress and very high distress for separate groupings of both income and ISRD quintiles.

**Number of 18–64 year olds**		**Greater capital city statistical areas ASGS 2011**							
		**Outcome: K10 high/very-high distress**				**Outcome: K10 very-high distress**			
		**Capital cities**		**Balance of state**		**Capital cities**		**Balance of state**	
		***n*** = **6,904**		***n*** = **5,428**		***n*** = **6,904**		***n*** = **5,428**	
**Australian population**		**OR (95% CI)**		**OR (95% CI)**		**OR (95% CI)**		**OR (95% CI)**	
Gender	Male	1.0*		1.0*		1.0*		1.0*
	Female	1.5	(1.3–1.9)	1.3	(1.0–1.8)	1.6	(1.1–2.3)	1.1	(0.7–1.8)
Age group (years)	18–24	1.0*		1.0*		1.0*		1.0*
	25–34	1.1	(0.8–1.4)	1.0	(0.6–1.6)	1.5	(0.8–2.9)	0.5	(0.2–1.5)
	35–44	1.0	(0.6–1.3)	1.2	(0.8–1.9)	1.3	(0.6–2.7)	1.1	(0.5–2.4)
	45–54	1.0	(0.7–1.3)	1.1	(0.7–1.7)	2.3	(1.1–4.7)	1.4	(0.7–2.8)
	55–64	0.7	(0.5–1.0)	0.9	(0.6–1.4)	1.3	(0.6–2.6)	0.9	(0.4–1.9)
Income quintiles^a^	(rich) 1	1.0*		1.0*		1.0*		1.0*
	2	1.6	(1.1–2.3)	0.7	(0.4–1.2)	1.7	(0.8–3.5)	0.5	(0.2–1.5)
	3	2.1	(1.3–2.9)	1.3	(0.9–2.1)	2.0	(1.0–4.2)	2.4	(0.9–6.2)
	4	3.7	(2.8–5.0)	3.1	(2.0–4.7)	5.3	(3.0–9.4)	4.5	(1.9–10.4)
	(poor) 5	6.1	(4.4–8.6)	5.8	(3.8–8.8)	8.2	(4.5–15.0)	8.6	(3.7–20.1)
	P for trend	<0.0001		0.04		<0.0001		0.04
ISRD quintiles^a^	(rich) 1	1.0*		1.0*		1.0*		1.0*
	2	1.3	(0.9–1.8)	1.1	(0.5–2.2)	2.0	(1.2–3.3)	1.2	(0.2–5.6)
	3	1.8	(1.4–2.4)	1.9	(1.2–3.2)	2.5	(1.4–4.7)	1.4	(0.4–5.5)
	4	2.2	(1.7–2.9)	2.0	(1.2–3.3)	2.9	(1.7–5.1)	1.5	(0.4–5.6)
	(poor) 5	2.7	(1.9–3.9)	2.6	(1.5–4.5)	3.6	(2.2–5.9)	2.7	(0.7–9.6)
	P for trend	<0.0001		<0.0001		<0.0001		<0.0001	

*The inclusion into the 4-variable multivariate model of interaction terms for the two socioeconomic variables (IRSD quintile and household income quintile) produced non-significant interactions and the magnitude of the Odds Ratios differ only minimally compared to the model without interaction terms*.

* *Reference category. IRSD, Index of Relative Socioeconomic Disadvantage; CI, confidence interval; ABS, Australian Bureau of Statistics; K10, Kessler 10. The K10 very-high distress category represents a score of 30 or higher. Combined high/very-high distress category represents a score of 22 or higher. CI, Confidence Intervals are based on replication-based standard error estimation. ABS weighting was used to produce population estimates*.

a *Analyses are adjusted for the effects of gender and age group*.

Lower socioeconomic status was associated with elevated distress in all areas of Australia, and elevated distress was more likely in those with lower household incomes. The corresponding concentration indices for very-high distress were −0.38 (−0.23, −0.53) in capital cities and −0.44 (−0.24, −0.64) outside capital cities; see Table 1.

Going against our third hypothesis, we found that the difference between rates of elevated distress in the richest two quintiles was greater than that of the lowest two quintiles for the Capital cities. In the Balance of state, however, the richest quintile had a higher rate of distress than the 2nd richest quintile. When examining income, the Rich/Poor Quintile Ratio in Australia in 2011/12 was 7.6 in capital cities and 7.2 outside capital cities. When examining very-high distress within income quintiles, the Rich/Poor Quintile Ratio was 7.2 in capital cities and 7.8 outside capital cities (see Table 1). Comparing the results showing the relative rates of elevated distress between the poorest and richest quintiles with the income ratios between the richest and poorest quintiles, it is interesting to find that these ratios are of a similar magnitude (in opposite directions). This empirical regularity has not been noted in previous Australian studies.

## Discussion

Overall, more than 1-in-4 people making up the poorest one-fifth of Australians have current psychological distress at a high/very-high level, and this compares to about 1-in-20 in the richest one-fifth of Australians. Our findings indicate that about 1-in-10 people making up the poorest one-fifth of Australians have very-high distress, and this reduces to <1-in-50 people in the richest one-fifth. These disparities are consistent both within and outside of capital cities. The national prevalence of elevated distress within income quintiles varies greatly, showing that household income is a strong predictor of elevated distress in Australia, with Poor/Rich Quintile Ratios of typically 4–5 for high/very-high levels and 7–8 for very-high levels. Another way to describe the level of inequality is to consider the typical income characteristics of 100 Australians with high level or greater distress. This shows that on average 40 Australians will come from the poorest income quintile, and only 8 will belong to the highest income quintile.

According to the report “Inequality in Australia, 2018,” 60% people who belong to the lowest 20% income group rely on social security for their income while those in the higher income groups rely mostly on earnings for their income (42). In addition, those who belong to the lowest 20% income group include single parents, people aged over 65 years, people who are unemployed, people born in non-English speaking countries, and people living outside capital cities (42). Financial hardship appears to be most common in these groups due to factors such as unsecured debt or unemployment (43) and can result in increased psychological distress.

There is evidence that increasing expenditure on mental health services is not succeeding in improving population mental health. While mal-distribution of care resources may be in part responsible for that, it also is possible that social and economic policies outside those directly involving mental healthcare may be adversely impacting population mental health as well as contributing to the disparities highlighted in this paper (44). Economic and social programmes aimed at poorer communities may promote community mental health (3, 45). For instance, simply increasing wages to align with costs of living has shown to improve psychological wellbeing irrespective of any differences in socioeconomic or demographic composition (46). So, increased psychological distress due to economic inequality is a social justice and human rights issue which, beyond concerns of direct mental health care provision, requires (1) Economic policies that promote financial security of populations, and funding for key services. (2) Labor policies that promote employment and protection against stress as well as (3) Education policies that provide quality basic education and cater for special needs (3). In addition, programs aimed at the prevention of mental disorders by utilizing a life span approach that targets risk factor modification might also be considered (47).

Prioritization of healthcare delivery toward poorer members of the community may also help to counter the influences of absolute poverty and relative disadvantage. GPs located where they are seeing more patients with lower incomes may be dealing with seven or more times the rate of mental-health related problems than those serving other patient groups with less low income households. These settings also often have fewer specialist services (21), making for great practical professional and personal challenges for the GP who is motivated toward such work. More should be done to develop and direct accessible and effective services to the poorer members of Australian society.

There are international calls to reduce health inequity (48) and indicators of inequity have received increased attention both globally (49, 50) and nationally (51). Australia's income and wealth inequality has shown a steady increase over the long term with the Gini index increasing from about 31% in the 1980s to 34% in 2010 (52). As discussed earlier, the increased use of antidepressants, increased mental health workforce and more psychological therapies has failed to reduce the prevalence of mental disorders in Australia (22, 44). National indicators that reflect inequalities in population mental health and highlight groups that are most in need are the need of the hour.

The different patterns of findings for income quintiles between Capital cities and Balance of state was unexpected. However, the pattern observed specifically in income quintiles 1–3 for Capital cities compared with Balance of state involves a number of over lapping confidence intervals and so we have not speculated on specific reasons for this.

Inequity associated with remoteness and socioeconomic status (21, 25, 53), or the effect of mental illness on labor market outcomes (54) have been described previously. However, these analyses rely on repeating the Australian National Survey on Mental Health and Wellbeing which is unlikely in the near future. It also involves repeating it in a comparable form, which did not happen between 1997 and 2007. Hence, indicators that rely on specifically targeted surveys may not be feasible. Existing indicators, which focus more on administrative and health service outputs, do not reflect broader socio-economic factors. New accountability indicators need to be developed that better reflect national health and social priorities and that are relevant to those who are most vulnerable to mental disorders (55).

A possible indicator that emerges from this paper could be the Poor/Rich quintile ratio (P/R QR) for the K10 scale high and very high band scores. Concentration indices and Gini coefficients are alternative indicators that have been extensively used and have a better theoretical basis. However, these are rather complex and not easily understood by all stakeholders. The most suitable indicator would be one that is easily understood and widely accepted. The P/R QR might be the preferable option as it is easily calculated from data obtained from a regularly conducted national survey, is easily understood and resonates with a wider audience. This index is used in other fields, for example as one of the main inequality indicators in the UNDP's well-known Human Development Reports. It is also necessary to determine targets for indicators as values or as trends; in this case a reduction in mental health inequality would show as a trend-decline in the P/R QR for high/very high K10 scores.

## Conclusions

This study highlights the substantial mental health inequality between the richer and poorer in Australia. This recommendation does not apply to a small group of people but rather operates progressively across a wide range of income levels. A key message for Australian policy makers responsible for public mental health and mental health care is the need for attention to, and accountability for, providing a more equitable distribution of both health and other services. Broader aspects of social and financial policy also would likely need adjustment to redress the prevailing determinants of inequity.

This study also concurs with others in calling for standard national mental health indicators and proposes the Poor/Rich quintile ratio (P/R QR) for high/very high K10 as a good indicator of equity of experience of mental health. This research is important and of global relevance as it addresses social justice aspects of mental health equity and furthers the evidence for more resources to be directed to the poor and disadvantaged in society.

## Data has been obtained from a third party

The data analyzed in this study was from the 2011/2012 National Health Survey and 2007 Survey of Mental Health and Wellbeing. It was obtained from the Australian Bureau of Statistics. Requests to access these datasets should be directed to the Australian Bureau of Statistics.

## Author contributions

This study was conceived by AI, JE, and GM. AI wrote the first draft. JE conducted the data analysis and wrote the methods section. BI reviewed the data analysis and suggested improvements. All authors reviewed manuscript drafts, suggested improvements, and approved the final draft.

### Conflict of interest statement

The authors declare that the research was conducted in the absence of any commercial or financial relationships that could be construed as a potential conflict of interest.

## References

[1] World Health Organization Mental Health Action Plan 2013-2020. Geneva (2013).

[2] Caron J Fleury MJ Perreault M Crocker A Tremblay J Tousignant M. Prevalence of psychological distress and mental disorders, and use of mental health services in the epidemiological catchment area of Montreal South-West. BMC Psychiatry (2012) 12:183. 10.1186/1471-244x-12-183 23110632 PMC3549723

[3] Patel V Lund C Hatherill S Plagerson S Corrigall J Funk M Mental disorders: equity and social determinants. In: eds Blas E Kurup A.S. Equity, Social Determinants and Public Health Programmes. Geneva: World Health Organization (2010).

[4] Starkey AJ Keane CR Terry MA Marx JH Ricci EM. Financial distress and depressive symptoms among African American women: identifying financial priorities and needs and why it matters for mental health. J Urban Health (2013) 90:83–100. 10.1007/s11524-012-9755-x 22930003 PMC3579302

[5] Selenko E Batinic B. Beyond debt. A moderator analysis of the relationship between perceived financial strain and mental health. Soc Sci Med. (2011) 73:1725–32. 10.1016/j.socscimed.2011.09.022 22019305

[6] Butterworth P Olesen SC Leach LS. The role of hardship in the association between socio-economic position and depression. Aust N Z J Psychiatry (2012) 46:364–73. 10.1177/0004867411433215 22508596

[7] Richardson T Elliott P Roberts R. The relationship between personal unsecured debt and mental and physical health: a systematic review and meta-analysis. Clin Psychol Rev. (2013) 33:1148–62. 10.1016/j.cpr.2013.08.009 24121465

[8] Jenkins R Bhugra D Bebbington P Brugha T Farrell M Coid J. Debt, income and mental disorder in the general population. Psychol Med. (2008) 38:1485–93. 10.1017/s0033291707002516 18184442

[9] Harper A Clayton A Bailey M Foss-Kelly L Sernyak MJ Rowe M. Financial health and mental health among clients of a community mental health center: making the connections. Psychiatric Serv. (2015) 66:1271–6. 10.1176/appi.ps.201400438 26234330

[10] Snowden LR. Poverty, safety net programs, and African Americans' mental health. Am Psychol. (2014) 69:773–81. 10.1037/a0037422 25486153

[11] Laliberte-Rudman D Yu B Scott E Pajouhandeh P. Exploration of the perspectives of persons with schizophrenia regarding quality of life. Am J Occupat Ther. (2000) 54:137–47. 10.5014/ajot.54.2.137 10732174

[12] Ljungqvist I Topor A Forssell H Svensson I Davidson L. Money and mental illness: a study of the relationship between poverty and serious psychological problems. Community Ment Health J. (2016) 52:842–50. 10.1007/s10597-015-9950-9 26433374

[13] Caron J Liu A. A descriptive study of the prevalence of psychological distress and mental disorders in the Canadian population: comparison between low-income and non-low-income populations. Chronic Dis Canada (2010) 30:84–94. 20609292

[14] Garratt EA Chandola T Purdam K Wood AM. The interactive role of income (material position) and income rank (psychosocial position) in psychological distress: a 9-year longitudinal study of 30,000 UK parents. Soc Psychiatry Psychiatric Epidemiol. (2016). 51:1361–72. 10.1007/s00127-016-1255-y 27376656

[15] Gornick JC Milanovic B Income inequality in the united states in cross-national perspective: redistribution revisited. In: LIS Center Research Brief (1/2015). New York: Luxembourg Income Study Center (2015). Available online at: https://www.gc.cuny.edu/CUNY_GC/media/CUNY-Graduate-Center/PDF/Centers/LIS/LIS-Center-Research-Brief-1-2015.pdf

[16] Pickett KE Wilkinson RG. Inequality: an underacknowledged source of mental illness and distress. Br J Psychiatry (2010) 197:426–8. 10.1192/bjp.bp.109.072066 21119145

[17] Cummins RA Personal income and subjective well-being: a review. J Happiness Stud. (2000) 1:133–58. 10.1023/A:1010079728426

[18] Parliament of Australia Medicare - Background Brief. Commonwealth of Australia (2004). Available online at: https://www.aph.gov.au/About_Parliament/Parliamentary_Departments/Parliamentary_Library/Publications_Archive/archive/medicare (Accessed Febuary 2, 2018).

[19] Slade T Johnston A Oakley Browne MA Andrews G Whiteford H. 2007 National Survey of Mental Health and Wellbeing: methods and key findings. Aust N Z J Psychiatry (2009) 43:594–605. 10.1080/00048670902970882 19530016

[20] Australian Government Department of Health,. MBS Online, Medicare Benefits Schedule. Commonwealth of Australia (2018). Available online at: http://www.mbsonline.gov.au/internet/mbsonline/publishing.nsf/Content/Downloads-201801 (Accessed February 2, 2018).

[21] Meadows GN Enticott JC Inder B Russell GM Gurr R Better access to mental health care and the failure of the Medicare principle of universality. Med J Aust. (2015) 202:297 10.5694/mja14.00330 25832148

[22] Jorm AF Patten SB Brugha TS Mojtabai R. Has increased provision of treatment reduced the prevalence of common mental disorders? Review of the evidence from four countries. World Psychiatry (2017) 16:90–9. 10.1002/wps.20388 28127925 PMC5269479

[23] Goldney RD Anne TW Marcus BA. Depression and remoteness from health services in South Australia. Aust J Rural Health (2007) 15:201–10. 10.1111/j.1440-1584.2007.00885.x 17542794

[24] Eckert KA Taylor AW Wilkinson DD Tucker GR. How does mental health status relate to accessibility and remoteness? Med J Aust. (2004) 181:540–3. 15540965 10.5694/j.1326-5377.2004.tb06442.x

[25] Enticott JC Meadows GN Shawyer F Inder B Patten S. Mental disorders and distress: associations with demographics, remoteness and socioeconomic deprivation of area of residence across Australia. Aust N Z J Psychiatry (2016) 50:1169–79. 10.1177/0004867415615948 26560843

[26] Kelly BJ Stain HJ Coleman C Perkins D Fragar L Fuller J. Mental health and well-being within rural communities: the Australian rural mental health study. Aust J Rural Health (2010) 18:16–24. 10.1111/j.1440-1584.2009.01118.x 20136810

[27] Butterworth P Handley TE Lewin TJ Reddy P Kelly BJ Psychological distress in rural Australia: regional variation and the role of family functioning and social support. J Public Health (2014) 22:481–8. 10.1007/s10389-014-0640-9

[28] Australian Health Ministers' Conference National Mental Health Policy/Australian Health Ministers. Canberra, ACT: Australian Government Publishing Service (1992).

[29] Commonwealth of Australia Response to Contributing Lives, Thriving Communities – Review of Mental Health Programmes and Services (2015). (Accessed March 13, 2016).

[30] Australian Institute of Health Welfare, Mental Health Indicators. AIHW (2016). Available online at: https://mhsa.aihw.gov.au/indicators/ (Accessed September 5, 2018).

[31] World Health Organization Global Reference List of 100 Core Health Indicators, 2015: Metadata. WHO (2015). Available online at: http://www.who.int/healthinfo/indicators/2015/metadata/en/ (Accessed August 8, 2018).

[32] Hickie I. Putting mental health services and suicide prevention reform into practice. Public Health Research Practice (2017) 27:e2721710. 10.17061/phrp2721710 28474047

[33] Andrews G Slade T. Interpreting scores on the Kessler Psychological Distress Scale (K10). Aust N Z J Public Health (2001) 25:494–7. 10.1111/j.1467-842X.2001.tb00310.x 11824981

[34] Kessler RC Andrews G Colpe LJ Hiripi E Mroczek DK Normand SL. Short screening scales to monitor population prevalences and trends in non-specific psychological distress. Psychol Med. (2002) 32:959–76. 10.1017/S0033291702006074 12214795

[35] Furukawa TA Kessler RC Slade T Andrews G. The performance of the K6 and the K10 screening scales for psychological distree in the Australian National Survey of Mental health and Well-Being. Psychol Med. (2003) 33:357–62. 10.1017/S0033291702006700 12622315

[36] Slade T Grove R Burgess P. Kessler psychological distress scale: normative data from the 2007 Australian National Survey of Mental Health and Wellbeing. Aust N Z J Psychiatry (2011) 45:308–16. 10.3109/00048674.2010.543653 21332432

[37] Australian Bureau of Statistics Australian Health Survey (2011/12): Users' Guide – Electronic (cat. no. 4363.0.55.001) (2013).

[38] Australian Bureau of Statistics 1216.0 – Australian Standard Geographical Classification (ASGC). ABS, (2011). Available online at: http://www.abs.gov.au/websitedbs/D3310114.nsf/home/Australian+Standard+Geographical+Classification+(ASGC) (Accessed July 16).

[39] Australian Bureau of Statistics 2033.0.55.001 - Census of Population and Housing: Socio-Economic Indexes for Areas (SEIFA), Australia 2011. ABS (2013a). Available online at: http://www.abs.gov.au/ausstats/abs@.nsf/mf/2033.0.55.001 (Accessed January 5, 2018).

[40] Australian Bureau of Statistics 4364.0.55.001 - National Health Survey: First Results, 2014-15. ABS (2015). Avilable onlibe at: http://www.abs.gov.au/AUSSTATS/abs@.nsf/Lookup/4364.0.55.001Explanatory%20Notes12014-15?OpenDocument (Accessed February 23, 2018).

[41] Mangham L,. ACT Consortium Guidance on Health Equity Analysis. ACT Consortium Core Group (2009). http://www.actconsortium.org/data/files/resources/80/Health-equityanalysis-ACT-Consortium-guidance.pdf (Accessed Febuary 2, 2018).

[42] Australian Council of Social Service and University of New South Wales. Inequality in Australia 2018. Strawberry Hills, NSW: Australian Council of Social Service and University of New South Wales (2018).

[43] Myles N Large M Myles H Adams R Liu D Galletly C. Australia's economic transition, unemployment, suicide and mental health needs. Aust N Z J Psychiatry (2017) 51:119–23. 10.1177/0004867416675035 27799415

[44] Meadows G Enticott J Rosenberg S Three Charts on: Why Rates of Mental Illness Aren't Going Down Despite Higher Spending. The Conversation Media Group (2018). https://theconversation.com/three-charts-on-why-rates-of-mental-illness-arent-going-down-despite-higher-spending-97534 (Accessed September 19).

[45] Kiely KM Leach LS Olesen SC Butterworth P. How financial hardship is associated with the onset of mental health problems over time. Soc Psychiatry Psychiatric Epidemiol. (2015) 50:909–18. 10.1007/s00127-015-1027-0 25683473

[46] Flint E Cummins S Wills J. Investigating the effect of the London living wage on the psychological wellbeing of low-wage service sector employees: a feasibility study. J Public Health (2014) 36:187–93. 10.1093/pubmed/fdt093 24014136

[47] National Mental Health Commission The National Review of Mental Health Programmes and Services. Sydney: National Mental Health Commission (2014).

[48] Cloninger CR Salvador-Carulla L Kirmayer LJ Schwartz MA Appleyard J Goodwin N. A time for action on health inequities: foundations of the 2014 Geneva Declaration on Person- and people-centered integrated health care for all. Int J Person Cent Med. (2014) 4:69–89. 26140190 PMC4485425

[49] Milanovic B Global Inequality: A New Approach for the Age of Globalization. Cambridge, MA: The President and Fellows of Harvard College (2016).

[50] Picketty T Translated by Goldhammer A Capital in the 21st Century. Cambridge, MA: The President and fellows of Harvard College (2014).

[51] Jericho G Damning evidence of wealth disparity highlights inequality across generations. The Guardian, Australia (2017). Available online at: https://www.theguardian.com/business/grogonomics/2017/aug/02/damning-evidence-of-wealth-disparity-highlights-inequality-across-generations

[52] The World Bank Gini Index (World Bank estimate). The World Bank (2017). Available online at: https://data.worldbank.org/indicator/SI.POV.GINI?end=2010&locations=AU&start=1981&view=chart (Accessed Febuary 2, 2018).

[53] Meadows G Burgess P Bobevski I. Distributing mental health care resources: strategic implications from the National Survey of Mental Health and Wellbeing. Aust N Z J Psychiatry (2002) 36:217–23. 10.1046/j.1440-1614.2002.01011.x 11982543

[54] Cornwell K Forbes C Inder B Meadows G. Mental illness and its effects on labour market outcomes. J Mental Health Policy Econ. (2009) 12:107–18. 19996474

[55] Rosenberg S Salvador-Carulla L. Perspectives: accountability for Mental Health: The Australian Experience. J Mental Health Policy Econ. (2017) 20:37–54. 28418836

